# Form of Lightning Rods

**Published:** 1875-08

**Authors:** 


The Form of Lightning Rods.—Knowing that electricity at rest always tends to diffuse itself on the surface, in fact, that it always *confines* itself to the surface, it became, at an early period, a question whether electricity in motion did not follow the same law. Pouillet determined the question in a very ingenious manner. He took a cylindrical wire of a certain size, and measured the resistance which it offered to a current of electricity. He then rolled the wire out flat, and measured its resistance again; it was found to be the same, although it is evident that the extent of the surface of the conductor was by this means greatly increased. Other experimenters have determined the question by different methods, but always with the same result. The committee of the French Academy, which included Becquerel, De la Rive, Pouillet and others, adopted a solid square bar as the best form for lightening-rods; and Sir William Snow Harris, though often quoted as favoring rods which present a large surface, says: "Provided the *quantity* of metal be present, the form under which we place it is evidently of no consequence to its conducting powers, since it would be absurd to suppose that a mass of metal, under any form, did not conduct electricity in all its particles; indeed, we know that it does so."—*Prof. John Phin, in Popular Science Monthly for August.*

